# Poisoning Children

**Published:** 1885-09

**Authors:** 


### Poisoning Children.

Forty years ago almost every mother thought her child must have paragoric or laudanum to make it sleep. These drugs will produce sleep, and a few drops, to many of them, will produce the sleep from which there is no waking. Many are the children who have been killed and whose health has been ruined for life by paragoric, laudanum and morphine, each of which is a narcotic product of opium. The use of opium in any form stupifies, intoxicates, and, in quite small quantities, kills. China resisted its importation by a war with Great Britain. In American and other countries, druggists are prohibited from selling either of the narcotics named to children at all, or to anybody, without labeling them "poison." Because children can be conveniently stupefied they have been forced into silence by a dose of narcotics although their stomachs might be full of indigestible food. The definition of "narcotic" is, "*A medicine which relieves pain and produces sleep, but which, in poisonous doses, produces stupor, coma, convulsions and death.*" As the dangerous nature of opium medicines began to be better understood, some dealers disguised the peculiar taste and smell in various mixtures, and thus continued to sell the same ingredients under the names of "Bateman's Drops," "Godfrey's Cordial," "Soothing Syrups," etc. Many substitutes for dangerous opium preparations have been searched for and experimented with ; the only effective and reliable one found was a composition of senna, pumpkin seed, oxygen, mint, anise seed, salt, soda and clarified sugar. This prescription met with pronounced favor on the part of pharmaceutical societies and medical authorities. It was used by physicians with results so gratifying that, subsequently, the originator of the formula procured for it, under the name of "Castoria," the protection of the Patent Offices of the United States and other governments. It is thus seen that the discovery of this remarkable and beneficent remedy was not an accident. While narcotic remedies can, with safety, be only administered by a physician, this prescription can be used by any one. The present extended use of Castoria is unquestionably the result of three facts: 1st. The indisputable evidence that it is harmless. 2d. That it not only allays stomach pains and quiets the nerves, but assimilates the food, and, 3d. It is an agreeable and perfect substitute for castor oil. This is a good deal for a medical journal to say. Our duty, however, is to expose danger and record the means of advancing health. The day for poisoning innocent children through greed or ignorance ought to end. To our knowledge, Castoria is a remedy which produces composure and health by regulating the system—not by stupefying it—and our readers are entitled to the information.

